# Current state of Ebola virus vaccines: A snapshot

**DOI:** 10.1371/journal.ppat.1010078

**Published:** 2021-12-09

**Authors:** Courtney Woolsey, Thomas W. Geisbert

**Affiliations:** 1 Galveston National Laboratory, University of Texas Medical Branch, Galveston, Texas, United States of America; 2 Department of Microbiology & Immunology, University of Texas Medical Branch, Galveston, Texas, United States of America; University of Kentucky, UNITED STATES

## Why are Ebola virus vaccines needed?

Four species of the genus *Ebolavirus* cause severe and often lethal disease in humans: *Sudan ebolavirus* (SUDV), *Bundibugyo ebolavirus* (BDBV), *Taï Forest ebolavirus* (TAFV), and *Zaire ebolavirus* (EBOV) [1]. Historically, EBOV has caused most *Ebolavirus* outbreaks and cases. The devastating 2013 to 2016 EBOV epidemic in West Africa, resulting in approximately 29,000 cases, prompted the global community to rapidly advance vaccine candidates that were previously in nascent stages of development [2]. The recent reemergence of EBOV in Guinea, Uganda, and the Democratic Republic of Congo (DRC) emphasizes the continued need for safe and effective vaccines against this deadly pathogen along with optimal deployment strategies. Heroic efforts by countless volunteers and organizations to vaccinate contacts of confirmed patients, healthcare staff, and frontline workers helped curb the West Africa epidemic and these more recent flare-ups [3,4]. As the global community was better prepared for the latter, a quick vaccination response was implemented that significantly abated transmission [4]. While EBOV outbreaks have historically impacted relatively small numbers of people on a global scale, they have caused great suffering and have inflicted an enormous economic toll in endemic countries. Demonstrated exportation of the virus to nonendemic regions [2,5] along with the bioweapon potential of ebolaviruses further warrant the development of EBOV vaccines worldwide.

## Which immunization strategy or combination of strategies is optimal for controlling Ebola virus infections?

The most effective vaccination strategies to prevent EBOV disease should balance benefits and risks to maximize vaccine impact while minimizing global costs, effort, and human suffering. Widespread mass vaccination is not considered an attainable goal as the endemic region includes much of West and Central Africa [2], putting over half of a billion individuals at risk. It is estimated that 80% vaccine coverage would be required to establish herd immunity against EBOV based on 90% vaccine efficacy and an estimated basic reproductive number value (R_0_; number of secondary cases that result from an individual infection) of 4 [5]. Financial/logistical hurdles and limited vaccine acceptance in the affected regions make such a vaccination rate a challenge. Ring vaccination (immunizing contacts (and contacts of contacts) of confirmed patients) similarly faces logistical barriers: Many EVD contacts cannot be reached or refuse vaccination, limiting its effectiveness [6]. Immunization of select groups also fuels equity concerns.

A tailored solution for each situation based on epidemiological characteristics and modeling is the best vaccine strategy. Early contact tracing along with ring vaccination may be adequate for isolated cases and small outbreaks, but supplemental approaches such as geographic- and/or population-based vaccination may be needed to curb large-scale outbreaks, especially if there is a high level of contact inaccessibility [5–7]. The latter vaccination strategies could additionally help foster vaccine trust. Routine immunization of healthcare and frontline workers in endemic regions as well as other specific groups (ambulance drivers, hospital cleaners, and burial teams) may also prove beneficial, as this population is at enhanced risk, and nosocomial transmission has been an amplifying factor in previous outbreaks [7].

## What is the current status, and what are some advantages and disadvantages, of the leading Ebola virus vaccines?

**Fig 1:**
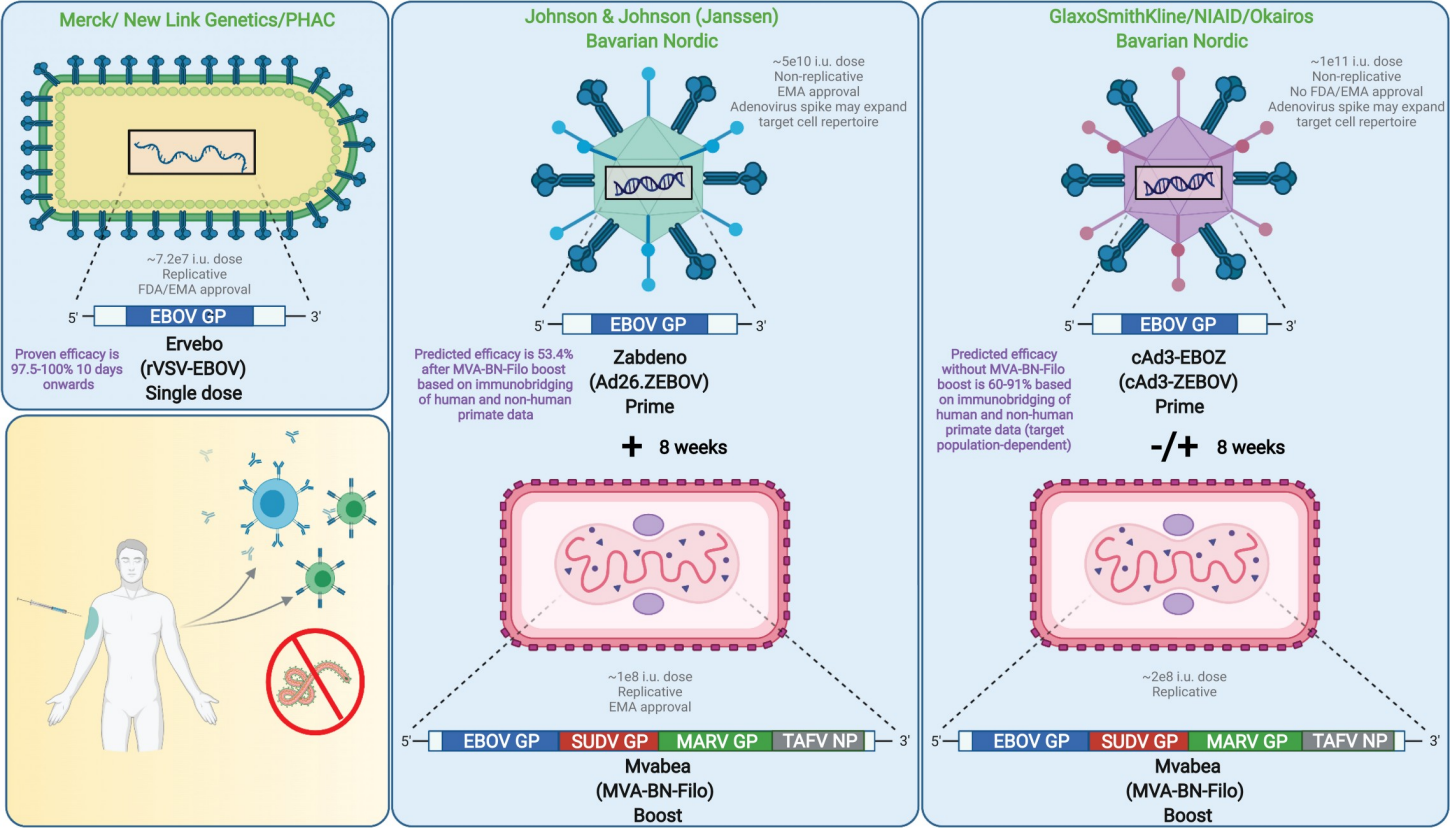
Leading EBOV vaccines. The most advanced vaccines in the US and Europe include Ervebo (rVSV-EBOV), Zabdeno/Mvabea (Ad26-ZEBOV/MVA-BN-Filo), and cAd3-EBOZ (with or without MVA-BN-Filo). These platforms use a viral vector to provoke an immune response, but, as illustrated, there are several distinctions among these 3 vaccines including vector virus, dose, efficacy, cell targets, and inclusion of a booster. Created with BioRender.com. Ad26, human adenovirus serotype 26; cAd3, chimpanzee adenovirus serotype 3; EBOV, Ebola virus (*Zaire ebolavirus*); EBOZ, Ebolavirus-Zaire species; EMA, European Medicines Agency; FDA, US Federal Drug Administration; GP, glycoprotein; i.u., infectious unit; MARV, Marburg virus; NIAID, National Immunology Allergy and Infectious Disease; NP, nucleoprotein; PHAC, Public Health Agency of Canada; rVSV, recombinant vesicular stomatitis virus; SUDV, Sudan virus; TAFV, Taï Forest virus; ZEBOV, Zaire ebolavirus.

Thirteen EBOV vaccine candidates have entered human clinical trials with 5 progressing to post-Phase I clinical trials [8]. Strengths and weaknesses for each of the 5 platforms are summarized in Table 1. The most advanced vaccines in the United States and Europe include Ervebo (rVSV-EBOV), Zabdeno/Mvabea (Ad26-ZEBOV/MVA-BN-Filo), and cAd3-EBOZ (Fig 1). All 3 platforms use a viral vector, or a modified version of a harmless surrogate virus, to provoke an immune response. Key benefits of virus-vectored vaccines are their ability to deliver antigen specifically to target cells and to induce robust, long-lived immunity. Ervebo, Zabdeno/Mvabea, and cAd3-EBOZ all express EBOV glycoprotein (GP) antigen to stimulate an immune response. GP is the sole surface protein of the EBOV virion and mediates attachment, fusion, and entry of target cells; thus, this protein serves as an attractive immunogen as it is readily recognized by the immune system and is the main target of the neutralizing antibody response [9]. Some general disadvantages of virus vaccine vectors include manufacturing obstacles, cold chain requirements, and difficulty in adapting to new virus variants.

**Table 1:** Advantages and disadvantages of post-Phase I clinical trial vaccines for EBOV disease.

Vaccine	Manufacturer	Advantages	Disadvantages	Status
Ervebo (rVSV-ZEBOV-GP; V920; rVSV**Δ**G-ZEBOV-GP) Monovalent, expresses EBOV GP (Kikwit variant)	Merck NewLink Genetics PHAC (National Microbiology Laboratory in Winnipeg, Manitoba)	• Only vaccine with proven clinical efficacy • Rapid immunostimulatory properties enable its use in an outbreak setting or as an emergency postexposure prophylactic • Single-dose approach eliminates the need for patient follow-up • Good safety profile, 2 SAEs reported deemed related to the vaccine (febrile reaction and anaphylaxis) that later resolved • Durable humoral immunity, strong immune responses reported at least 2 years after vaccination • Lower doses of vaccine needed than adenovirus-based vaccines	• Only targets EBOV, which was responsible for the 2013–2016 outbreaks and more recent flare-ups • Only licensed for adults ≥18 years of age • Reports of arthritis in a subset of vaccinees associated with increasing age and increased IgG titers beyond 6 months • Infectious virus found in synovial joints of vaccinees suggests unlikely but possible vaccine shedding/secondary transmission • Requires ≥60°C storage temperature; −60°C to −80°C stability is 36 months, 2°C to 8°C for no more than 2 weeks, room temperature for no more than 4 hours	• Licensed by US FDA and EMA • Granted Breakthrough Therapy • Designation by the US FDA and PRIME status by the EMA • Phase III trials completed in Africa, the US, Canada, and Europe • Expanded access protocols used in Guinea and in the DRC • Tested in children older than or equal to 1 year (PREVAC), women that later became pregnant, and HIV–positive individuals; appears immunogenic and safe but still examining its suitability in these populations • Durability, antibody threshold of protection? • Safety and immunogenicity in the immunocompromised and pregnant/lactating women?
Zabdeno/Mvabea (Ad26.ZEBOV + heterologous MVA-BN-Filo boost) Multivalent after second dose, Zabdeno expresses EBOV GP (Mayinga) Mvabea expresses EBOV GP, SUDV GP, TAFV NP, and MARV GP	Johnson & Johnson (Janssen division) Bavarian Nordic	• Approved for individuals 1 year and older • Good safety profile, 2 SAEs reported deemed related to vaccine (Miller Fisher syndrome and small fiber neuropathy) that later resolved • Multivalent after second dose; targets EBOV, SUDV, and TAFV as well as MARV (although, only indicated for EBOV) • Replication deficiency eliminates vaccine shedding concerns • Multiple storage options: Ad26.EBOV: −20°C to −60°C for 48 months and +2 to +8°C for 12 months; MVA-BN-Filo: 20°C to −60°C for 42 months and +2 to +8°C for 6 months	• Lower predicted vaccine efficacy than Ervebo (approximately 53%) based on stringent nonhuman primate bridging data • Requires 2 doses (patient follow-up cause for concern) • Not ideal for outbreak settings as 8 weeks must pass before the second dose is administered • High doses of vaccine required for immunogenicity compared to Ervebo • Booster vaccination recommended 4 months post second dose • Mvabea does not include immunogen targeting *Bundibugyo* or *Bombali ebolaviruses* • Data on cross-protection against non-EBOV or MARV does not exist • Preexisting immunity to vector may reduce the effectiveness of the vaccine	• Licensed by EMA under exceptional circumstances • Phase I/II/III trials completed in Europe, the US, and Africa • Submitted dossier to the US FDA to request licensure using the Animal Rule • Submitting to WHO for EUAL • Other vaccine combination/variants are being explored to enhance immunogenicity/efficacy of Zabdeno and Mvabea • Durability, antibody threshold of protection? • Safety and immunogenicity in the immunocompromised and pregnant/lactating women?
ChAd3-EBOZ with or without Mvabea (cAd3-ZEBOV; ChAd3-EBO-Z) Monovalent, expresses EBOV GP (Mayinga variant) Mvabea expresses EBOV GP, SUDV GP, TAFV NP, and MARV GP	GlaxoSmith Kline Okairos NIAID	• Single-dose and/or optional multivalent boost • Good safety profile, no SAE reports, mild-to-moderate reactogenicity • Can be administered to children (1 year and older) and adults • Uses chimpanzee-specific adenovirus to circumvent preexisting immunity to vector • Replication deficiency eliminates vaccine shedding concerns • At high dose (1e11 particles), can be used for reactive vaccination	• Lower predicted vaccine efficacy than Ervebo (approximately 60%–90% protection with high 1e11 dose no Mvabea boost based on nonhuman primate bridging data) • chAd3-EBOZ only targets EBOV • Optional Mvabea targets more virus species but is only indicated for EBOV • Higher doses of vaccine required for immunogenicity compared to Ervebo • Requires ≥60°C storage temperature for single-dose vials (stability at ≤60°C is 24 months), currently evaluating stability at other storage conditions • Antibody responses decreased by roughly half at 180 days after vaccination; booster recommended	• Not yet licensed by the US FDA or EMA • Phase II trials completed in Europe, the US, and Africa • Ongoing trials to explore safety and immunogenicity of other vaccine variations including multivalent, homologous, and heterologous combinations as well as shorter dosing intervals • Completed randomized, double-blind Phase II trial in adults: immediate vs. placebo + delayed (6 months) vaccination for adults • Completed randomized, observer blind Phase II trial in children: immediate Vx + Placebo (Meningococcal Vx) at 6 mo vs. Immediate placebo + Vx at month 6 for children • Durability with booster, antibody threshold of protection? • Immunogenicity in immunocompromised and HIV populations?
Ad5-EBOV Monovalent, expresses EBOV GP (Makona variant)	BIT CanSino (China)	• Single dose • Good safety profile, no SAE reports; adverse reactions mild and self-limiting • Storage at +2°C to +8°C for 12 months (2 vials of lyophilized powder + 1 vial of diluent)	• Only targets EBOV • Preexisting immunity to Ad5 vector may reduce the effectiveness of the vaccine • Only indicated for 18 to 60 years of age • No clinical efficacy data, only immunogenicity data • GP-specific antibodies decreased 85% at day 168	• Not licensed in the US, UK, or EU • Licensed in China based on Animal Rule by the Chinese Food and Drug Administration • Submitting to WHO for Emergency Use • Phase II—Assessment and Listing (EUAL) • Durability, antibody threshold of protection? • Immunogenicity in immunocompromised and HIV populations?
GamEvac-Combi and GamEvacLyo Heterologous prime-boost w/ rVSV and Ad5 expressing EBOV GP (Makona)	Gamaleya Research Institute of Epidemiology and Microbiology (Russia)	• Combo approach to take advantage of benefits of each platform (consists of rVSV and Ad5 expressing EBOV GP) • Stable at −16°C to −20°C for 12 months	• Only targets EBOV • 2 doses (prime + boost at 21 days) • Only indicated for 18 to 55 years • Preexisting immunity to Ad5 vector may reduce the effectiveness of the vaccine • No published clinical efficacy data, only immunogenicity data • Preexisting neutralizing Ad5 antibodies negatively influenced GP responses in half-dose but not the full-dose group	• Not licensed in the US, UK, or EU • Licensed by the Ministry of Health of the Russian Federation for emergency use in December 2015 based on Phase I and II safety and immunogenicity data • Completed Phase III trial in Guinea (Kindia) • Completed Phase IV trial in Russia • Durability, antibody threshold of protection? • Immunogenicity in immunocompromised and HIV populations?

Ad26, human adenovirus serotype 26; BIT, Beijing Institute of Technology; cAd3, chimpanzee adenovirus serotype 3; DRC, Democratic Republic of Congo; EBOV, Ebola virus; EBOZ, Ebolavirus-Zaire species; EMA, European Medicines Agency; EUAL, Emergency Use Authorization Listing; FDA, US Federal Drug Administration; GP, glycoprotein; HIV, human immunodeficiency virus; MARV, Marburg virus; NIAID, National Immunology Allergy and Infectious Disease; NP, nucleoprotein; rVSV, recombinant vesicular stomatitis virus; SAE, serious adverse event; SUDV, Sudan virus; TAFV, Taï Forest virus; Vx, Vaccination; WHO, World Health Organization; ZEBOV, Zaire ebolavirus.

Modified from reference [8] Table 1 (Ebola Vaccine Team B/CIDRAP/WellcomeTrust report “Completing the Development of Ebola Vaccines”).

## Ervebo (rVSV-EBOV; V920)

Ervebo is a live-attenuated, replication-competent, single-dose vaccine originally developed and shown to completely protect nonhuman primates (NHPs) by scientists at the Public Health Agency of Canada and the US Army [10]. The recombinant vesicular stomatitis virus (rVSV)-based vaccine expresses a functional full-length EBOV GP instead of the native VSV GP (rVSV-EBOV), thereby narrowing host tropism specifically toward cell targets of EBOV. Despite decades of promising preclinical research in NHPs, clinical development of rVSV-EBOV stalled until the 2013 to 2016 West African epidemic. In the face of the looming crisis, Phase I/II clinical trials were conducted in healthy volunteers in Europe, Africa, and the US in 2014 [8]. A single dose of Ervebo was shown to be highly immunogenic in volunteers, producing robust humoral responses in nearly all recipients. EBOV GP–specific antibody responses were strong after 2 years, indicating that Ervebo is also durable [11]. Adverse effects in vaccinees were typically mild [8].

Following safety and immunogenicity testing, Ervebo was deployed in Guinea for Phase III efficacy evaluation in 2015. Results indicated that the vaccine was “100% effective” as no new cases were identified in the vaccinated population 10 days or more after immunization [3]. As Ervebo proved a resounding success in an outbreak setting, over 300,000 contacts were immunized with Ervebo during the 2018 to 2020 DRC EBOV outbreak [4,5]. According to preliminary results, the vaccine was 97.5% effective at stopping EBOV transmission compared to no vaccination [4]. The vaccine also has potential as an emergency postexposure prophylactic as demonstrated by its use in a laboratory incident [12] as well as several NHP studies [13]. Currently, Ervebo is the only vaccine with proven clinical efficacy and US Food and Drug Administration (FDA) approval. Preexisting immunity to the rVSV vector is of little concern given the low seropositivity in the general population and because immune responses are predominantly directed at the VSV GP, which is absent from the vaccine. For example, previous vaccination of NHPs with a Lassa virus GP precursor-expressing rVSV vaccine did not abrogate immunity when NHPs were sequentially immunized with an EBOV GP–expressing rVSV vaccine and challenged with EBOV [14]. An ultra-cold chain requirement for long-term storage is a disadvantage for Ervebo.

## Zabdeno/Mvabea (Ad26-ZEBOV/MVA-BN-Filo)

The Zabdeno/Mvabea vaccine employs both AdVac technology and MVA-BN technology and is delivered in 2 doses: Zabdeno (Ad.26.ZEBOV) is administered first, and Mvabea (MVA-BN-Filo) is given approximately 2 months later [8]. Hence, this preventive 2-dose regimen is not suitable for an outbreak response where immediate protection is necessary.

The Zabdeno component is derived from adenovirus serotype 26 (Ad26) and expresses the EBOV GP in place of the replication-essential adenovirus early 1 region [15]. Unlike Ervebo, Zabdeno is unable to replicate in humans. While this attribute may be desirable for a preventive vaccine due to its perceived safety profile, much higher doses are required to elicit a protective immune response (approximately 72 million infectious unit (i.u.) dose of Ervebo versus 50 billion i.u. for Zabdeno). The adenovirus surface spike protein is retained in contrast to Ervebo, which expands the target cell repertoire. Because Ad26 is associated with human disease, many individuals may have preexisting immunity against the virus vector (10% to 90% depending on geographic location), particularly in EBOV-endemic regions [16]. Nevertheless, immune responses of Zabdeno/Mvabea-vaccinated subjects were not markedly different between seronegative individuals and those exhibiting baseline Ad26 seropositivity [17]; it is not known whether a subsequent dose or exposure to the same Ad26 vector backbone will impact vaccine effectiveness.

The Mvabea component consists of a modified Vaccinia Ankara virus (MVA) encoding GPs from EBOV, SUDV, and Marburg virus (MARV), and TAFV nucleoprotein [15]. A multivalent filovirus vaccine targeting multiple *Ebolavirus* species such as Mvabea is optimal for preventive administration.

While protective efficacy against MARV and other *Ebolavirus* species has not yet been demonstrated, preclinical studies indicate a Zabdeno/Mvabea prime-boost immunization provided full protection of NHPs against an EBOV challenge [18]. Phase I/II/III clinical trials have demonstrated that Zabdeno/Mvabea is safe and elicits strong neutralizing and nonneutralizing antibody responses in vaccine recipients along with both CD4+ and CD8+ T cell responses [15,17]. During the 2018 to 2020 outbreak, Zabdeno/Mvabea was administered to over 20,000 individuals, with 9,560 receiving the second dose [19]. A population-level Phase III trial evaluating the efficacy of Zabdeno/Mvabea was initiated in DRC, but that data have not yet been reported [16]. Due to the absence of efficacy data, predictive efficacy of Zabdeno/Mvabea for European Medicines Agency (EMA) approval was based on bridging clinical immunogenicity data with efficacy and immunogenicity data in NHPs [15]. The highest protective efficacy for Zabdeno/Mvabea in NHPs was found at a dosing interval of 8 weeks. When given an identical clinical dose at this interval, all animals survived. Briefer dosing intervals provided less protection: 80% at 42 days and 50% to 57% at 28 days. Based on the pooled immunogenicity data from healthy adults, the mean predicted survival probability was estimated at 53.4%. Humoral responses are largely sustained for 2 years but decline to levels >10-fold lower than peak titers. Boosters at approximately 1 year induced anamnestic responses with an approximate 12- to 55-fold increase in EBOV GP binding antibody titers within a week [15]. As the protective threshold of circulating antibodies is not established, one cannot ascertain the level needed to protect humans from EBOV. Nonetheless, all or most boosted NHPs survived when challenged 1.5 years post-primary vaccination. A Zabdeno booster is recommended for individuals at high risk of Ebola virus exposure if their 2-dose vaccine regimen was completed more than 4 months ago.

## cAd3-EBOZ (chAd3-EBO Z) with or without MVA-BN-Filo

cAd3-EBOZ/MVA-BN-Filo was developed by NIAID/NIH in collaboration with Okairos [8]. The vaccine platform is similar to Zabdeno/Mvabea; however, the first dose consists of an attenuated chimpanzee adenovirus (cAd3). This feature addresses issues associated with preexisting immunity to the vector such as the case with Zabdeno. An optional heterologous booster of multivalent Mvabea is administered as a subsequent dose. Phase I/II clinical trials proved the vaccine to be well tolerated and immunogenic [20–22]. Most adverse events were self-limited and mild, indicating that the vaccine is safe. Strong humoral responses were noted in vaccinees, particularly after the Mvabea booster. Four weeks after immunization with the CAd3 vaccine alone, GP-specific antibody responses were slightly lower yet similar to those induced by Ervebo, but less durable at 12 months [23]. Neutralization antibody activity and injection site reactions were also similar between the 2 vaccines. Human and NHP bridging data predicted protection of 91% of Malian and 60% of US participants given a single high 1 × 10^11^ infectious particle dose of cAd3-EBOZ based on reciprocal binding antibody levels; a titer of 1,000 or higher is hypothesized to confer high-level protection. A subsequent dose of Mvabea stimulated anamnestic antibody and CD4/CD8 T-cell responses, suggesting that this booster might further boost protection and duration of immunity [23].

## What are some other vaccines in clinical development?

CanSino and GamEvac vaccines—platforms derived from rVSV- and adenovirus-based technologies—are currently licensed for emergency use in China and the Russian Federation, respectively (Table 1) [24,25]. Another promising EBOV Phase I vaccine candidate is an additional rVSV vector, Vesiculovax (Auro Vaccines), which expresses EBOV GP along with a highly attenuated form of VSV GP [26,27]. Other EBOV vaccine candidates where Phase I trial data have been published include a 2-dose DNA vaccine targeting EBOV [28], a bivalent DNA plasmid vaccine targeting EBOV and MARV [29,30], and a 2-dose monovalent nanoparticle recombinant EBOV GP vaccine [31].

## Are there remaining challenges for Ebola virus vaccines?

While substantial progress has been made in the development of EBOV vaccines, multiple questions remain unanswered including the following: (1) what is the durability and the immediacy of immune responses generated by different vaccines; (2) what are the specific correlates and thresholds of protection; (3) do any interactions or interferences exist between vaccines and potential therapeutics; (4) what is the safety of these vaccines in special populations, particularly pregnant women and the immunocompromised; and (5) can vaccines be formulated to be stable for long-term storage at 2 to 8°C, which would be useful in endemic areas [8]?

Another major hurdle is mitigating the economic risks for manufacturers and distributors of EBOV vaccines since the demand may not be high enough to warrant stockpiling. Outbreaks also tend to occur in resource-poor countries leaving little financial incentive for commercial development. In January 2021, the International Coordinating Group (ICG) comprised of public and private benefactors established an Ebola vaccine stockpile in Sweden with the goal of manufacturing 500,000 doses. Stockpiling is an important step toward controlling EBOV outbreaks as it is critical for ensuring timely access to vaccines for at-risk populations [32].

Lastly, the most advanced vaccines are solely indicated for protection against one species of *Ebolavirus*: EBOV. Although multivalent vaccines (monovalent cocktails or vaccine vectors expressing various GPs) have and are being developed, no studies have specifically evaluated immune responses or efficacy against other *Ebolavirus* species (e.g., SUDV, BDBV, TAFV). Future work should focus on the development of vaccines that confer protection across all medically relevant species of the genus *Ebolavirus*, bearing in mind that intraspecies mutations may also arise that impact vaccine effectiveness.
